# Machine learning in predicting *T*-score in the Oxford classification system of IgA nephropathy

**DOI:** 10.3389/fimmu.2023.1224631

**Published:** 2023-08-04

**Authors:** Lin-Lin Xu, Di Zhang, Hao-Yi Weng, Li-Zhong Wang, Ruo-Yan Chen, Gang Chen, Su-Fang Shi, Li-Jun Liu, Xu-Hui Zhong, Shen-Da Hong, Li-Xin Duan, Ji-Cheng Lv, Xu-Jie Zhou, Hong Zhang

**Affiliations:** ^1^ Renal Division, Peking University First Hospital, Kidney Genetics Center, Peking University Institute of Nephrology, Key Laboratory of Renal Disease, Ministry of Health of China, Key Laboratory of Chronic Kidney Disease Prevention and Treatment, Peking University, Ministry of Education, Beijing, China; ^2^ Hunan Provincial Key Lab on Bioinformatics, School of Computer Science and Engineering, Central South University, Changsha, China; ^3^ WeGene, Shenzhen Zaozhidao Technology, Shenzhen, China; ^4^ Shenzhen WeGene Clinical Laboratory, Shenzhen, China; ^5^ Department of Pediatrics, Peking University First Hospital, Beijing, China; ^6^ Institute of Medical Technology, Health Science Center of Peking University, Beijing, China; ^7^ The Sichuan Provincial Key Laboratory for Human Disease Gene Study, Research Unit for Blindness Prevention of Chinese Academy of Medical Sciences (2019RU026), Sichuan Academy of Medical Sciences and Sichuan Provincial People’s Hospital, University of Electronic Science and Technology of China, Chengdu, China

**Keywords:** IgA nephropathy, machine learning, Oxford classification system, prediction model, end-stage kidney disease

## Abstract

**Background:**

Immunoglobulin A nephropathy (IgAN) is one of the leading causes of end-stage kidney disease (ESKD). Many studies have shown the significance of pathological manifestations in predicting the outcome of patients with IgAN, especially *T*-score of Oxford classification. Evaluating prognosis may be hampered in patients without renal biopsy.

**Methods:**

A baseline dataset of 690 patients with IgAN and an independent follow-up dataset of 1,168 patients were used as training and testing sets to develop the pathology *T*-score prediction (*T*_pre_) model based on the stacking algorithm, respectively. The 5-year ESKD prediction models using clinical variables (base model), clinical variables and real pathological *T*-score (base model plus *T*_bio_), and clinical variables and *T*_pre_ (base model plus *T*_pre_) were developed separately in 1,168 patients with regular follow-up to evaluate whether *T*_pre_ could assist in predicting ESKD. In addition, an external validation set consisting of 355 patients was used to evaluate the performance of the 5-year ESKD prediction model using *T*_pre_.

**Results:**

The features selected by AUCRF for the *T*_pre_ model included age, systolic arterial pressure, diastolic arterial pressure, proteinuria, eGFR, serum IgA, and uric acid. The AUC of the *T*_pre_ was 0.82 (95% CI: 0.80–0.85) in an independent testing set. For the 5-year ESKD prediction model, the AUC of the base model was 0.86 (95% CI: 0.75–0.97). When the *T*_bio_ was added to the base model, there was an increase in AUC [from 0.86 (95% CI: 0.75–0.97) to 0.92 (95% CI: 0.85–0.98); *P* = 0.03]. There was no difference in AUC between the base model plus *T*_pre_ and the base model plus *T*_bio_ [0.90 (95% CI: 0.82–0.99) *vs*. 0.92 (95% CI: 0.85–0.98), *P* = 0.52]. The AUC of the 5-year ESKD prediction model using *T*_pre_ was 0.93 (95% CI: 0.87–0.99) in the external validation set.

**Conclusion:**

A pathology *T*-score prediction (*T*_pre_) model using routine clinical characteristics was constructed, which could predict the pathological severity and assist clinicians to predict the prognosis of IgAN patients lacking kidney pathology scores.

## 1 Introduction

Immunoglobulin A (IgA) nephropathy (IgAN) is one of the most common forms of glomerulonephritis worldwide. The clinical manifestations are heterogeneous, ranging from asymptomatic proteinuria or microscopic hematuria to rapid deterioration in kidney function (1). It was reported that approximately 20%–30% of patients with IgAN would progress to kidney failure within 20 years (2). Therefore, early identification of high-risk patients with IgAN prone to ESKD is beneficial for early intervention in delaying disease progression. Great endeavors have been taken by many researchers to search for the risk factors for developing ESKD in patients with IgAN. Generally accepted risk factors affecting the progression of IgAN included decreased glomerular filtration rate (GFR), 24-h proteinuria >1 g/day, hypertension, and renal pathological manifestations (3–9). These risk factors have been used to build various scoring models for predicting the prognosis of IgAN based on traditional statistical methods (4, 10–14). However, these scoring models are constructed by the small sample sizes and different pathological scoring criteria, which may affect the accuracy and generalization of these scoring models. Moreover, the interactions between the characteristics and their effect on ESKD, the non-linear relationship among predictors, and the effects of therapeutic regimens make the interpretation of the data more complicated.

Machine learning, as a branch discipline of artificial intelligence, has obvious advantages in processing high-dimensional and sparse data. Machine learning algorithms can learn the relationship between input features and target outcomes as well as the relationship between features through a large amount of training data. Several studies have successfully constructed ESKD prediction models for patients with IgAN through machine learning algorithms (15–20). By comparing the performance of traditional statistical methods and different machine learning algorithms in predicting ESKD or halving of estimated glomerular filtration rate from baseline, Chen et al. showed that the XGBoost algorithm performed best (16). XGBoost, as a machine learning algorithm, assembles the weak prediction models to construct a prediction model (16, 21). Several studies have tried to construct event prediction models for a specific clinical outcome based on the XGBoost algorithm (22, 23). However, no matter whether it was a traditional prediction formula or a machine learning-based predictive model in IgAN, pathology scores showed consistently significant weighting among many parameters (15, 16, 19, 24). In 2009, the Oxford classification, an international consensus, was proposed to classify IgA nephropathy based on histopathological features to predict its prognosis and guide clinical treatment. The revised Oxford classification in 2017 divided IgAN into five categories, namely, “(1) mesangial hypercellularity (M); (2) endocapillary hypercellularity (E); (3) segmental glomerulosclerosis (S); (4) tubular atrophy/interstitial fibrosis (T); (5) cellular/fibrocellular crescents (C)” (25), which were shown to be the independent predictors in predicting renal outcome (24, 26). Since 2009, over 20 validation studies have tried to prove the predictive value of the MEST scores in some retrospective cohorts of patients with IgAN, which provided consistent evidence that the mesangial hypercellularity (M), segmental glomerulosclerosis (S), and tubular atrophy/interstitial fibrosis (T) each reliably provided prognostic value by univariate analysis (26), but T lesion was suggested to be the strongest predictor of renal survival. Hernan et al. summarized the results of these studies and found that M was of independent prognostic value in 5 out of 19, E in 4 out of 19, S in 7 out of 19, and T in 13 out of 19 (26). The *C*-score was adopted in the revised classification system in 2017, and three of the five prognostic studies on IgA nephropathy showed that *C*-score was associated with poor prognosis (26–28). In the constructed IgAN prognosis prediction models, it was observed that the T lesions showed greater weight in predicting prognosis compared with many other clinical and pathological parameters (14, 16). For example, in the prognosis prediction model constructed by Chen et al., there were three indexes that can be integrated to predict ESKD, namely, *T*, global sclerosis, and urine protein, among which the *T*-score ranked first in the weight of importance (16). However, the *T*-score is derived from the kidney biopsy, an invasive manipulation, sometimes refused by patients and cannot be repeated in clinical routine for detecting disease progression. Hence, it is of great significance to explore whether pathological T lesions can be predicted by the patient’s clinical variables at the same time.

The purposes of our study are 1) to construct a pathology *T*-score (*T*_pre_) prediction model based on the patient’s clinical variables at the same time which may be able to predict whether there is a pathological T lesion and 2) to evaluate whether the predicted T can be used to assist in predicting ESKD.

## 2 Methods

### 2.1 Study participants

This study had two independent datasets. Dataset 1, a baseline dataset without follow-up data, comprised 690 patients with IgAN. These patients received the kidney biopsy in our center but returned to local for follow-up. Dataset 2, a follow-up dataset (PKU-IgAN cohort), included 1,808 patients with IgAN who were registered and with long-term follow-up in the Peking University First Hospital IgAN database from 1997 to 2020 (29). All patients with IgAN were diagnosed based on the histologic and immunofluorescence study of the renal biopsy, and those with <8 glomeruli per biopsy section were excluded (29). After excluding 243 patients without blood lipid data, 28 patients presented at younger than 16 years of age, and 14 patients presented acute kidney failure, 1,523 patients in dataset 2 were finally enrolled in this study, consisting of 1,168 patients with Oxford MEST-C scores and 355 patients lacking Oxford MEST-C scores.

**Figure 1:**
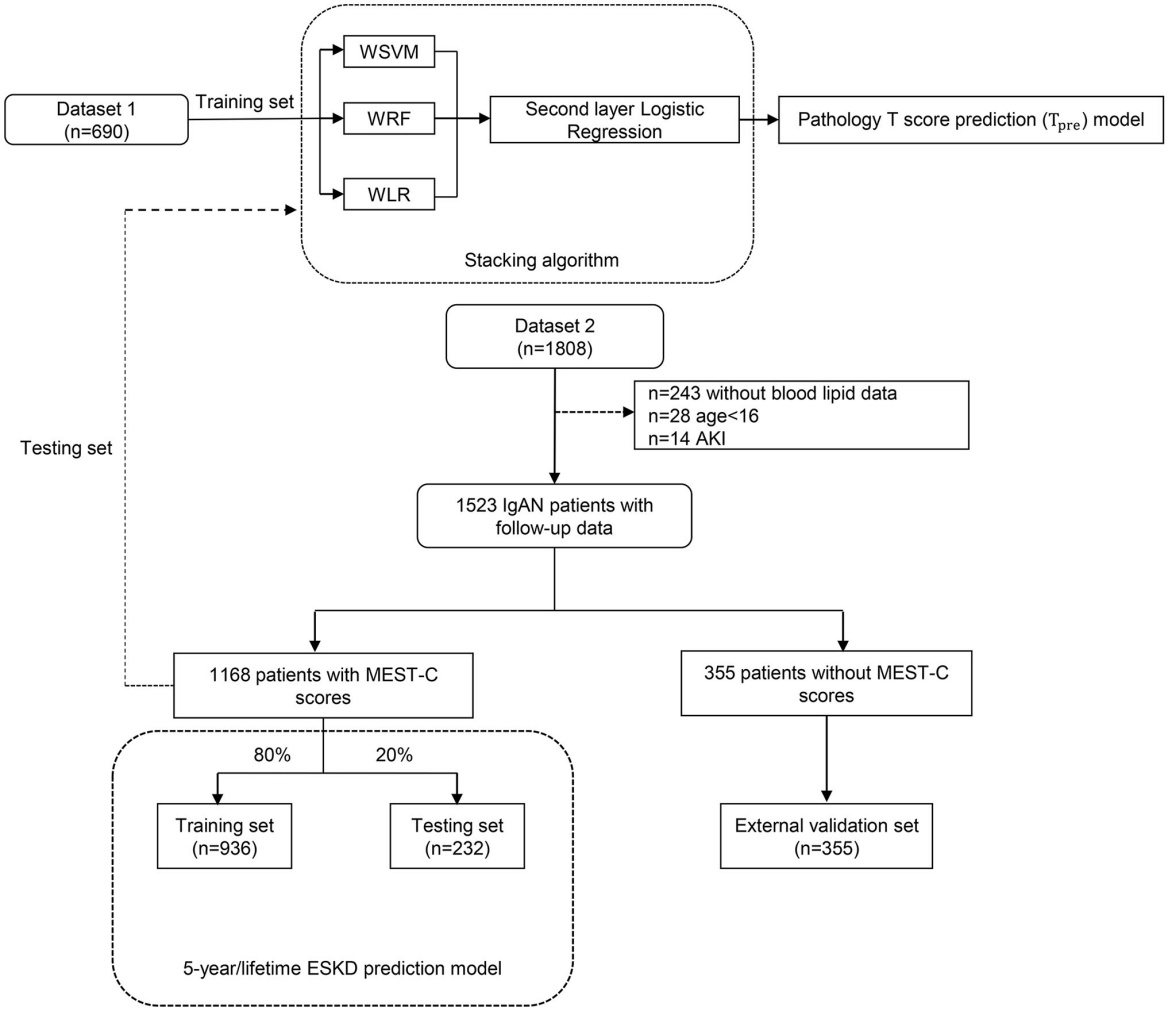
The flowchart of this study. WSVM, weighted support vector machine; WRF, weighted random forest; WLR, weighted logistic regression; AKI, acute kidney injury.

Finally, a total of 690 patients in dataset 1 and 1,168 patients with Oxford MEST-C scores in dataset 2 were enrolled in our study as the modeling group, and 355 patients without Oxford MEST-C scores in dataset 2 were enrolled in this study as the external validation group (**Figure 1**).

All clinical characteristics were collected at the time of the renal biopsy. The estimated glomerular filtration rate (eGFR) was calculated using the Chronic Kidney Disease Epidemiology Collaboration (CKD-EPI) formula (30). Renal biopsies were categorized according to established criteria for the Oxford MEST-C scoring system (24, 26, 31). Mean arterial pressure (MAP, mm Hg) was defined as diastolic pressure plus a third of the pulse pressure. The end-stage kidney disease (ESKD) was defined as eGFR <15 ml/min/1.73 m^2^, dialysis, or kidney transplantation. Our study was approved by the Ethics Committee of Peking University First Hospital (IRB number 2020Y197). Written informed consent was provided by all participants.

### 2.2 Pathology *T*-score prediction model

The pathology *T*-score prediction (*T*_pre_) model, constructed by the stacking algorithm, was used to predict whether IgAN patients would have T lesions (yes or no). The stacking algorithm is an integrated machine learning algorithm that can summarize several models and predict new observations. It utilizes the prediction of a collection of models as input for training a second-level model. This second-level model aims to find the best combination of the prediction of first-level models. Stacking can shield the capabilities of a range of well-performing models so that a better output prediction model can be achieved (32). In our study, we combined three machine learning algorithms, namely, support vector machine (SVM), random forest (RF), and logistic regression as first-level models, and then logistic regression as the second-level model to output the final probability of the binary *T*-score (with or without tubular atrophy/interstitial fibrosis, *T*_pre_).

The input variables used in this model were chosen by AUCRF (33), a method using the random forest to find the optimal set for prediction. Variables entered into the AUCRF included age, sex, body mass index, systolic arterial pressure, diastolic arterial pressure, mean arterial pressure, hypertension, eGFR, proteinuria, microhematuria, history of gross hematuria, serum IgA, serum uric acid, serum triglycerides, total cholesterol, high-density lipoprotein, and low-density lipoprotein.

### 2.3 Five-year ESKD prediction model

Several studies have demonstrated the value of tubular atrophy/interstitial fibrosis (T) in predicting ESKD in patients with IgAN (16, 19, 24, 34, 35). To evaluate whether the predicted *T*-score could help predict ESKD and how effective it was, we constructed a 5-year ESKD prediction model based on the XGBoost algorithm. To illustrate the significance of tubular atrophy/interstitial fibrosis in predicting ESKD, we first constructed a 5-year ESKD prediction model with only clinical variables as input variables (base model). Then, the 5-year ESKD prediction model using clinical variables and the real pathological T lesions score (*T*_bio_, T0 was assigned 0, T1 and T2 were assigned 1) was also developed (base model plus *T*_bio_) to evaluate the additive value of atrophy/interstitial fibrosis (T) in predicting ESKD. Finally, to evaluate whether the value of *T*_pre_ in predicting ESKD of patients with IgAN was consistent with real pathological T lesions (*T*_bio_) when the base model plus *T*_bio_ was trained in the training set, the *T*_bio_ of the testing set was replaced by the corresponding *T*_pre_ predicted by the pathology *T*-score prediction model and then the testing set was used to evaluate the model performance (the base model plus *T*_pre_). For the base model plus *T*_pre_, the purpose of training the model using real pathological *T*-score (*T*_bio_) was for the model to learn the true value of T for predicting ESKD.

XGBoost is a kind of ensemble of the decision tree, whose advantages include higher-order interactions and complex non-linear relationships between the model features and the outcome (21). It has been shown to achieve impressive performance in predicting renal failure risk and provide explanations for variables by ranking their importance (16, 34). We also applied other machine learning algorithms to our data set for evaluating whether the predicted T could be used in ESKD prediction models based on different algorithms, including RF, penalized regression, artificial neural network (ANN), and SVM.

Characteristics selected by the Cox proportional hazards model were collected at the time of the renal biopsy at enrollment [age, sex, systolic arterial pressure, diastolic arterial pressure, proteinuria, eGFR, serum IgA, serum uric acid, serum triglycerides, total cholesterol, low-density lipoprotein, and history of previous use of renin–angiotensin system (RAS) inhibitors and immunosuppressants as well as pathological T lesions], whereas the binary outcome (ESKD within 5 years after diagnostic kidney biopsy, yes or no) represented the output data. For these variables, we imputed missing values to the means for continuous characteristics and the mode for categorical characteristics. Because of missing information on serum triglycerides, total cholesterol, and low-density lipoprotein in some cases, 243 patients without blood lipid data were excluded to avoid inaccuracy due to missing value filling (**Figure 1**).

To confirm that the *T*_pre_ can be used in the ESKD prediction model at multiple levels, we also constructed a lifetime ESKD prediction model based on XGBoost. The process and approach were the same as building the 5-year ESKD prediction model. The primary outcome was time-to-event ESKD. The survival time for the kidney without ESKD event was calculated from the kidney biopsy to the last follow-up.

The XGBoost was allowed to generate boosting trees at most 110 times, and the maximum depth of each tree was constrained to 5. To avoid overfitting, we further set the L2 regularization term on weights as 1 and stop training if the performance did not improve by more than 15 rounds. At last, the optimal prediction model parameters and architectures were selected by the five-fold cross-validation.

The patients of dataset 2 without Oxford MEST-C scores combined with the corresponding *T*_pre_ were used as an additional external validation set to evaluate the performance of the ESKD prediction model using *T*_pre_.

### 2.4 Statistical analysis

The sociodemographic and clinical variables were calculated and expressed as the mean ± standard deviation for variables with approximately symmetrical distributions and as median (interquartile range 25th–75th percentile) for variables with skewed distribution. All categorical variables are expressed as frequencies and percentages. Univariate analyses based on the Cox proportional hazards model (36) were conducted to evaluate the association between the baseline clinical characteristics and ESKD event. Clinical characteristics associated with ESKD event in univariate analysis (*P* < 0.05) or if they were clinically relevant were used as input features of the 5-year ESKD prediction model.

For predicting 5-year ESKD status (yes or no) and *T*-score (0 or 1), the performance of the models was assessed by calculating the accuracy, sensitivity, specificity, and area under the receiver operating characteristic (ROC) curve (AUC). For predicting lifetime ESKD risk, we quantify the performance of the model by concordance statistic (*C*-statistic), which is a general concept of the area under the curve (AUC) for time-to-event survival data (37). The *C*-statistic compares the rank of predicting probability and the rank of the survival time in the real world. The calibration ability of the models was assessed by the Hosmer–Lemeshow test and calibration scatter plot, in which *P*-value >0.05 indicated no very significant difference between the predicted probability predicted by the model and the true outcome frequencies during a certain time period. SPSS version 26.0 software and R 3.6.3 were used for the statistical analysis. All *P*-values were two-tailed, and *P <*0.05 was considered statistically significant.

## 3 Results

### 3.1 Characteristics of the study participants

The clinical characteristics of 690 patients with IgAN in dataset 1 are shown in **Table 1**. The mean age of these patients was 32.38 ± 11.32 years at the time of renal biopsy. The male-to-female ratio was 1.2:1. The mean arterial pressure was 94.44 ± 14.02 mm Hg. The median value of eGFR was 84.66 (range, 63.32–107.50) ml/min per 1.73 m^2^, and daily proteinuria was 1.38 (range, 0.66–2.89) g/day.

**Table 1:** Baseline characteristics of patients with IgAN enrolled in this study to construct the pathology *T*-score prediction model at the time of kidney biopsy.

Characteristics	Training set	Testing set	*P*-value
	(dataset 1)	(dataset 2 with MEST-C scores)	
Patients (*n*)	690	1,168	
Age at biopsy, years	32.38 ± 11.32	35.10 ± 11.73	1.00 × 10^−6^
Sex (male/female)	370/320	583/585	0.12
Systolic blood pressure, mm Hg	124.77 ± 18.28	123.67 ± 15.09	0.18
Diastolic blood pressure, mm Hg	79.28 ± 13.11	78.54 ± 11.00	0.22
Mean arterial pressure, mm Hg	94.44 ± 14.02	93.59 ± 11.42	0.17
eGFR, ml/min per 1.73 m^2^	84.66 (63.32–107.50)	85.91 (60.94–107.23)	0.69
Proteinuria, g/day	1.38 (0.66–2.89)	1.27 (0.66–2.45)	0.10
Serum IgA level, g/l	3.13 ± 1.21	3.29 ± 1.20	0.01
Uric acid, μmol/l	347.10 ± 114.95	367.63 ± 101.86	1.52 × 10^−4^
Triglycerides, mmol/l	1.61 (1.10–2.38)	1.62 (1.07–2.42)	0.64
Total cholesterol, mmol/l	4.70 (3.99–5.61)	4.77 (4.02–5.67)	0.23
Low-density lipoprotein, mmol/l	2.71 (2.12–3.33)	2.75 (2.23–3.38)	0.19
Renal biopsy, *n*/*n* (%)			
Mesangial (M) 1	560/690 (81.16%)	461/1,168 (39.47%)	3.37 × 10^−68^
Endocapillary (E) 1	128/690 (18.55%)	400/1,168 (34.25%)	4.23 × 10^−13^
Glomerular sclerosis (S) 1	225/690 (32.61%)	733/1,168 (62.76%)	3.33 × 10^−36^
Tubulointerstitial damage (T1+T2)	182/690 (26.38%)	392/1,168 (33.56%)	1.00 × 10^−3^

Data are expressed as mean ± SD, median (interquartile range), absolute, and percent frequency.

IgAN, immunoglobulin A nephropathy; eGFR, estimated glomerular filtration rate.

For the 1,168 follow-up patients with Oxford MEST-C scores in dataset 2, the mean age was 35.10 ± 11.73 years at the time of renal biopsy. The male-to-female ratio was 1:1. The mean arterial pressure was 93.59 ± 11.42 mm Hg. The eGFR was 85.91 (range, 60.94–107.23) ml/min per 1.73 m^2^, and daily proteinuria was 1.27 (range, 0.66–2.45) g/day (**Table 1**). For the variables used to train the pathology *T*-score prediction (*T*_pre_) model, there were no statistically significant differences in clinical parameters between dataset 1 and dataset 2 except for age (32.38 ± 11.32 *vs*. 35.10 ± 11.73, *P* = 1.00 × 10^−6^), serum IgA level (3.13 ± 1.21 *vs*. 3.29 ± 1.20, *P* = 0.01), and serum uric acid level (347.10 ± 114.95 *vs*. 367.63 ± 101.86, *P* = 1.52 × 10^−4^). Among these, 158 patients (13.53%) had reached the event of ESKD during the median 67.5-month follow-up. The unadjusted hazard ratios (HRs) between the different variables and ESKD are reported in **Table 2**. The risk of ESKD significantly increased for every 10.0 mm Hg increase in the MAP [HR: 1.34, 95% confidence interval (CI): 1.18–1.53, *P* = 1.10 × 10^−5^] and increased for every 1.0 g/day in the daily proteinuria (HR: 1.10, 95% CI: 1.05–1.15, *P* = 1.60 × 10^−5^). For each ml/min per 1.73 m^2^ decrease in eGFR, the risk of ESKD increased by 4% (HR: 0.96, 95% CI: 0.96–0.97, *P* = 1.24 × 10^−27^). For each mg/dl increase in uric acid, the risk of ESKD increased by 38% (HR: 1.38, 95% CI: 1.29–1.49, *P* = 1.47 × 10^−19^). Moreover, there was the strongest association between the risk of ESKD and the presence of tubulointerstitial lesions (HR: 3.34, 95% CI: 2.73–4.07, *P* = 1.72 × 10^−32^).

**Table 2:** Risk estimated by Cox proportional hazard model for ESKD in patients of dataset 2 with Oxford MEST-C scores.

Risk factor	Non-ESKD (*n* = 1,010)	ESKD (*n* = 158)	*P*-value	HR (95% CI)
Age, years	35.21 ± 11.84	34.41 ± 11.02	0.55	1.00 (0.98–1.01)
Male (%)	482 (47.72%)	101 (63.92%)	1.96 × 10^−4^	1.85 (1.34–2.57)
Systolic arterial pressure, mm Hg	123.00 ± 14.70	128.02 ± 16.77	3.00 × 10^−6^	1.02 (1.01–1.03)
Diastolic arterial pressure, mm Hg	78.13 ± 10.66	81.18 ± 12.71	2.88 × 10^−4^	1.03 (1.01–1.04)
Mean arterial pressure, mm Hg	93.09 ± 11.06	96.80 ± 13.10	1.10 × 10^−5^	1.03 (1.02–1.04)
Proteinuria, g/day	1.17 (0.61–2.28)	1.99 (1.15–3.56)	1.60 × 10^−5^	1.10 (1.05–1.15)
eGFR, ml/min per 1.73 m^2^	89.13 (66.05–110.14)	53.69 (37.47–85.39)	1.24 × 10^−27^	0.96 (0.96–0.97)
Serum IgA level, g/l	3.30 ± 1.22	3.17 ± 0.99	0.27	0.93 (0.81–1.06)
Uric acid, μmol/l	358.08 ± 97.12	429.22 ± 110.27	1.47 × 10^−19^	1.01 (1.00–1.01)
Triglycerides, mmol/l	1.59 (1.06–2.37)	1.85 (1.13–2.69)	0.01	1.12 (1.03–1.23)
Total cholesterol, mmol/l	4.77 (4.03–5.66)	4.76 (3.99–5.84)	0.72	1.02 (0.93–1.11)
Low-density lipoprotein, mmol/l	2.75 (2.24–3.36)	2.75 (2.19–3.59)	0.77	1.02 (0.90–1.16)
Renal biopsy				
M0/M1	636/374 (62.97%/37.03%)	71/87 (44.94%/55.06%)	2.00 × 10^−6^	2.14 (1.56–2.93)
E0/E1	668/342 (66.14%/33.86%)	100/58 (63.29%/36.71%)	0.36	1.16 (0.84–1.61)
S0/S1	402/608 (39.80%/60.20%)	33/125 (20.89%/79.11%)	2.10 × 10^−5^	2.31 (1.57–3.39)
T0/T1+T2	723/287 (71.58%/28.42%)	53/105 (33.54%/66.46%)	1.72 × 10^−32^	3.34 (2.73–4.07)
C0/C1+C2	421/589 (41.68%/58.32%)	54/104 (34.18%/65.82%)	1.00 × 10^−3^	1.47 (1.17–1.86)
Therapy				
Renin–angiotensin system blocks	960 (95.05%)	151 (95.57%)	0.14	0.57 (0.26–1.21)
Corticosteroids/cytotoxic drugs	474 (46.93%)	107 (67.72%)	3.60 × 10^−5^	2.02 (1.45–2.82)
Follow-up, months	67.50 (37.75–105.25)	67.50 (38.00–97.25)		

Data are expressed as mean ± SD, median (interquartile range), absolute, and percent frequency.

ESKD, end-stage kidney disease; CI, confidence interval; HR, hazard ratio; eGFR, estimated glomerular filtration rate.

### 3.2 Performance of the pathology *T*-score prediction model

**Figure 2:**
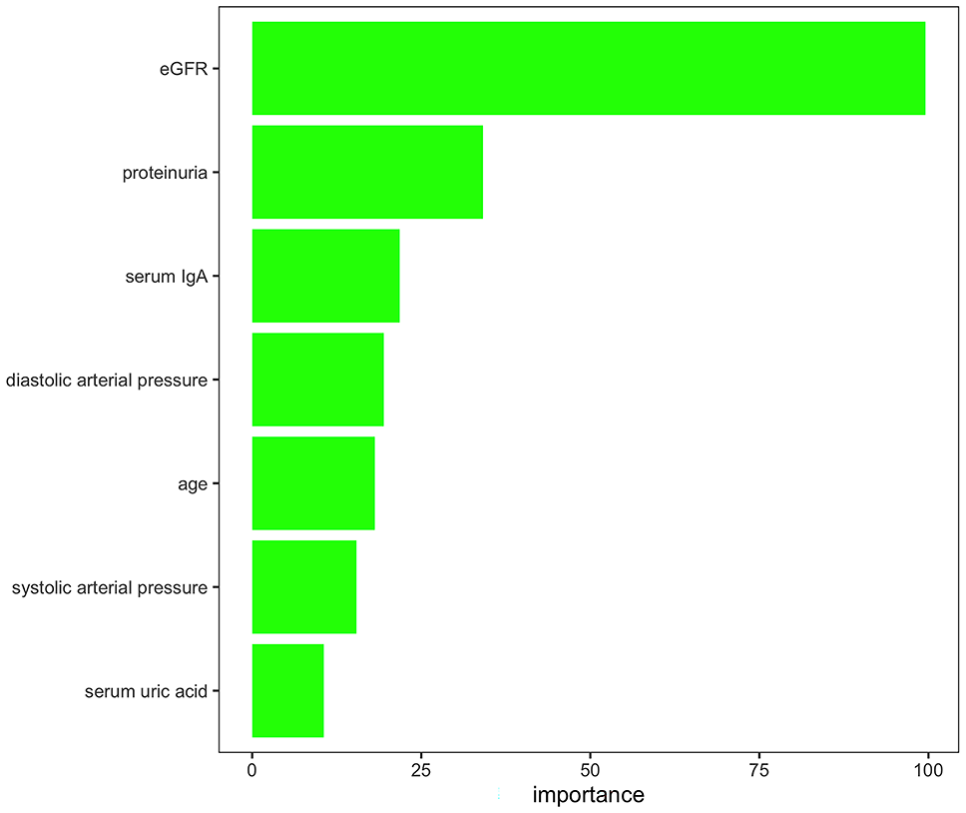
Variables selected by AUCRF for the pathology *T*-score prediction model. The importance scores of the clinical variables with a probability of selection higher than 0.7 in repeated cross-validation of the optimal random forest model to predict the presence or absence of T lesions.

Feature reductions were conducted using the AUCRF algorithm, which was used to select the optimal random forest model with the least number of predictive variables to predict the presence or absence of T lesions. Clinical variables with a probability of selection higher than 0.7 were selected in repeated cross-validation of the optimal random forest model (optimal AUC = 0.82). Finally, the features selected by AUCRF for the T prediction model included age, systolic arterial pressure, diastolic arterial pressure, proteinuria, eGFR, serum IgA, and uric acid (**Figure 2**). The 690 IgAN patients with Oxford MEST-C scores in dataset 1 as the training set were taken to develop a pathology *T*-score prediction model. The 1,168 IgAN patients with Oxford MEST-C scores in dataset 2 as the testing set were used only for reporting the performance of the model and were not used for development or fine-tuning.

**Figure 3:**
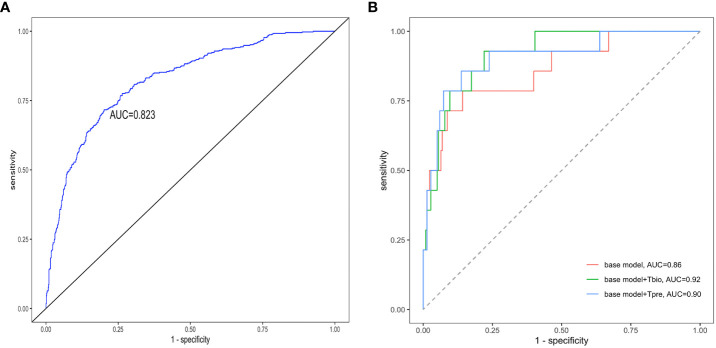
Receiver operating characteristic curves of the prediction models. The receiver operating characteristic curves for **(A)** the pathology *T*-score prediction (*T*_pre_) model and **(B)** the 5-year ESKD prediction model. The base model was the 5-year ESKD prediction model based on the XGBoost algorithm with only clinical variables as input variables. The base model + *T*_bio_ was the 5-year ESKD prediction model based on XGBoost using clinical variables and the real pathological T lesions score (*T*_bio_, T0 was assigned 0, and T1 and T2 were assigned 1). The base model + *T*_pre_ was when the base model plus *T*_bio_ was trained using clinical variables and *T*_bio_, and the *T*_bio_ of the testing set was replaced by the corresponding *T*_pre_ predicted by the pathology *T*-score prediction model. The clinical variables used for the 5-year ESKD prediction model included age, sex, systolic arterial pressure, diastolic arterial pressure, proteinuria, eGFR, serum IgA, uric acid, triglycerides, total cholesterol, low-density lipoprotein, and history of previous use of renin–angiotensin system (RAS) inhibitors and immunosuppressants. AUC, area under the curve.

If a predictive model has an AUC of higher than 0.75, it will be considered to have a good discriminating ability. The pathology T prediction model achieved a discrimination of 0.82 (95% CI: 0.80–0.85) [area under the receiver operating characteristic (ROC) curve (AUC)] in the testing set (**Figure 3A**). The ROC curve had 0.74 sensitivity and 0.77 specificity, which indicated that it had better clinical utility.

### 3.3 Performance of the 5-year ESKD prediction model

The unadjusted Cox regression analysis suggested that sex, systolic arterial pressure, diastolic arterial pressure, proteinuria, eGFR, uric acid, triglycerides, and tubular atrophy/interstitial fibrosis (T) were risk factors for developing ESKD (**Table 2**). A study supported elevated serum IgA as a causal factor in IgA nephropathy through Mendelian randomization (38). Some studies have suggested the association between the poor prognosis of renal disease and dyslipidemia. Higher triglycerides and cholesterol levels have been proven to be independent risk factors for the progression of kidney disease (39). Hence, clinical variables (age, sex, systolic arterial pressure, diastolic arterial pressure, proteinuria, eGFR, serum IgA, uric acid, triglycerides, total cholesterol, low-density lipoprotein, history of previous use of RAS inhibitors and immunosuppressants) and the pathology T lesions (*T*_bio_, T0 was assigned 0, T1 and T2 were assigned 1) were used as the input variables of the 5-year ESKD prediction model.

To make the predictive model achieve a good performance, the 1,168 follow-up IgAN patients with Oxford MEST-C scores in dataset 2 were randomly divided into training and testing sets at a ratio of 8:2. The training set included 936 patients and the testing set included 232 patients. The training set was used to perform five-fold cross-validation to select the optimal prediction model. The testing set was used to assess the performance.

**Figure 4:**
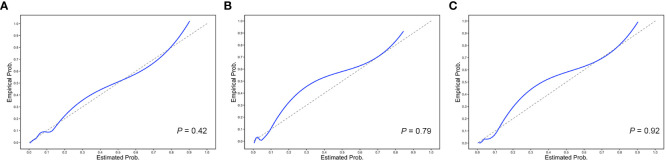
Calibration plots of the 5-year ESKD prediction models. The calibration plots for **(A)** the base model, **(B)** the base model plus *T*_bio_, and **(C)** the base model plus *T*_pre_. The *P*-values for the Hosmer–Lemeshow test of the base model, the base model plus *T*_bio_, and the base model plus *T*_pre_ were 0.42, 0.79, and 0.92, respectively, which indicated that these models had a good calibration.

The performance value of the 5-year ESKD prediction model using only the above clinical variables as input variables (base model) was 0.86 (95% CI: 0.75–0.97) in the test set (**Figure 3B**). To test whether the *T*_bio_ could improve the predictive performance of the 5-year ESKD prediction model, we added *T*_bio_ to the base model. An increase in AUC [from 0.86 (95% CI: 0.75–0.97) to 0.92 (95% CI: 0.85–0.98); *P* = 0.03] showed a better discriminating ability, which indicated that the T was important for judging the prognosis of patients with IgAN (**Figure 3B**). To test whether *T*_pre_ had a similar effect on judging the prognosis of IgAN patients, after training the 5-year ESKD prediction model with the training set, we replaced the *T*_bio_ in the testing set with the corresponding *T*_pre_ to see the discrimination effect. The AUC was 0.90 (95% CI: 0.82–0.99) in the testing set (**Figure 3B**). The performance of the base model plus *T*_pre_ did not differ from that of the base model plus *T*_bio_ [AUC for the base model plus *T*_pre_ 0.90 (95% CI: 0.82–0.99) *vs*. AUC for the base model plus *T*_bio_ 0.92 (95% CI: 0.85–0.98), *P* = 0.52, **Table 3**], which showed that the value of the *T*_pre_ in predicting the ESKD of patients was comparable to that of *T*_bio_. The calibration of the three prediction models is shown in **Figures 4A–C**. The *P*-values for the Hosmer–Lemeshow test of the base model, the base model plus *T*_bio_, and the base model plus *T*_pre_ were 0.42, 0.79, and 0.92, respectively, which indicated that these models had a good calibration. These results suggested the importance of T in predicting ESKD, and *T*_pre_ can be used to assist clinicians in assessing the prognosis of patients without pathology reports.

**Table 3:** Performance comparison for the prediction on 5-year ESKD status with different predictors in the testing subset.

Model	Accuracy	Sensitivity	Specificity	AUC
Clinical variables	0.85	0.79	0.86	0.86
Clinical variables plus *T*_bio_	0.83	0.93	0.78	0.92
Clinical variables plus *T*_pre_	0.86	0.86	0.86	0.90

The clinical variables include age, sex, systolic arterial pressure, diastolic arterial pressure, proteinuria, eGFR, serum IgA, uric acid, triglycerides, total cholesterol, low-density lipoprotein, and history of previous use of renin–angiotensin system (RAS) inhibitors and immunosuppressants.

T_bio_, the real pathological T-score quantified as either 0 (absent) or 1 (T1 or T2); T_pre_, the pathological T-score predicted by the baseline pathology T-score prediction (T_pre_) model.

**Table 4** shows the performance of the 5-year ESKD prediction model based on different machine learning algorithms in the testing set using *T*_pre_. All models have good prediction performance, which indicated that *T*_pre_ could be used in ESKD predictive models built on different algorithms.

**Table 4:** Performance of the 5-year ESKD prediction model using *T*_pre_ based on different machine learning algorithms in the testing set.

Model	Accuracy	Sensitivity	Specificity	AUC
XGBoost	0.86	0.86	0.86	0.90
Random forest	0.82	0.79	0.87	0.89
Penalized regression	0.80	0.93	0.80	0.88
Artificial neural network	0.78	0.86	0.77	0.86
Support vector machine	0.71	0.86	0.62	0.77

The model was trained using clinical variables and the T_bio_, and the T_bio_ was replaced with the corresponding T_pre_ predicted by the pathology T-score prediction model in the test subset.

For the lifetime ESKD prediction model based on XGBoost using only clinical variables (base model), the *C*-statistic was 0.82 (95% CI: 0.80–0.84) in the testing set. The discriminating ability of the base model plus *T*_pre_ was also comparable to the base model plus *T*_bio_ [*C*-statistic: 0.85 (95% CI: 0.83–0.86) *vs*. 0.85 (95% CI: 0.83–0.86), *P* = 0.11] in the testing set.

### 3.4 External validation of the ESKD prediction model using *T*_pre_

The 355 patients without MEST-C scores in dataset 2 were included as the external validation population for evaluating the performance of the 5-year ESKD prediction model. Because patients did not have MEST-C scores, the *T*_pre_ predicted by the pathology *T*-score prediction model was used in the 5-year ESKD prediction model. The AUC of the 5-year ESKD prediction model using *T*_pre_ based on XGBoost was 0.93 (95% CI: 0.87–0.99). We listed the AUC of the applied other machine learning algorithms in **Table 5**.

**Table 5:** Performance of the 5-year ESKD prediction model using *T*_pre_ based on different machine learning algorithms in the external validation set.

Model	Accuracy	Sensitivity	Specificity	AUC
XGBoost	0.82	1.00	0.81	0.93
Logistic regression	0.72	1.00	0.71	0.90
Artificial neural network	0.50	1.00	0.48	0.79
Support vector machine	0.87	0.67	0.87	0.74
Random forest	0.86	0.83	0.86	0.92

The characteristics used in the basic model include age, gender, SBP, DBP, eGFR, IgA, UTP, UA, TG, TCHO, LDL, history of corticosteroids/cytotoxic drugs, and renin–angiotensin system blockers.

In the lifetime ESKD prediction model using *T*_pre_, the *C*-statistic was 0.92 (95% CI: 0.90–0.94). We have shown here that both models have a good performance in the external validation set, indicating the reliability of *T*_pre_ for assisting in evaluating the prognosis of IgAN.

## 4 Discussion

We developed a pathology *T*-score prediction (*T*_pre_) model that can predict whether the patient with IgAN may have tubulointerstitial lesions at this time based on clinical variables when the patient did not undergo a renal biopsy or did not want to repeat the renal biopsy for progression assessment. We further constructed the 5-year/lifetime ESKD prediction model based on the XGBoost algorithm to confirm the importance of T in predicting ESKD, and *T*_pre_ can replace the real pathological T lesions for assisting clinicians in evaluating the prognosis of IgAN patients without pathology reports. In addition, the ESKD prediction model built based on different machine learning algorithms had good discriminating ability by using clinical variables and *T*_pre_, which indicated the reliability and universality of *T*_pre_ for assisting in evaluating the prognosis of IgAN.

For developing the pathology *T*-score (*T*_pre_) prediction model, we first used the AUCRF algorithm to select the clinical variables that may be associated with the tubulointerstitial lesions. Feature selection before training the predictive model can prevent dimensional disaster, reduce training time, prevent overfitting, enhance model generalization ability, and enhance the understanding of features and feature values, which also determines the upper limit of the effect of a machine learning task. The AUCRF is based on the RF algorithm, which is used for feature reduction based on optimizing the area under the ROC curve (AUC) of the random forest (33). It was found that age, systolic arterial pressure, diastolic arterial pressure, proteinuria, eGFR, serum IgA, and uric acid may be the clinical characteristics associated with tubular atrophy/interstitial fibrosis. Mechanism studies are needed to explore the inherent causality of these correlations and predictive capability. There have been reports indicating the association between reduced initial eGFR, higher initial MAP, proteinuria, and tubular atrophy/interstitial fibrosis (31). Next, we used the stacking algorithm to construct the pathology *T*-score prediction (*T*_pre_) model based on the clinical characteristics selected by the AUCRF. A single learner has over- or underfitting problems, and to obtain a learner with excellent generalization performance, we can train multiple individual learners to form a strong learner through a certain combination strategy. This method of integrating multiple individual learners is called ensemble learning. Stacking is one of the methods of ensemble learning. The advantage of integration is that different models can learn different features of the data, and the results after fusion tend to perform better (40). As our results showed, when we used an independent dataset as the testing set, the AUC of the pathological *T*-score prediction (*T*_pre_) model reached 0.82, which indicates the good discriminating ability of this *T*_pre_ prediction model.

A host of studies have indicated that pathological T lesions play an important role in predicting prognosis (14, 35, 41). At the same time, most current ESKD prediction models based on different methods or algorithms all include pathology *T*-score (14, 16, 19). Nevertheless, a renal puncture is invasive, which may cause a series of complications and has a host of contraindications, such as severe hypertension, coagulation disorders, solitary kidney, and so on (42). Furthermore, the number of patients at high risk of renal puncture may increase in the near future because of the aging of the population and the increased use of anticoagulant medication (43). For the patients who lack the report of kidney biopsy or do not want to undergo repeat renal puncture for disease progression assessment and evaluation of the effect of drug therapy, the clinician could not assess the prognosis of these patients with IgAN by using the established ESKD prediction model. The pathology *T*-score prediction (*T*_pre_) model we developed may solve this problem. We also constructed a 5-year/lifetime ESKD prediction model based on XGBoost to assess whether the value of *T*_pre_ in predicting ESKD of patients with IgAN was consistent with real pathological *T*-score. The performance of the base model plus *T*_pre_ was similar to the base model plus *T*_bio_, which showed that the *T*_pre_ can replace the real pathological *T*-score for prognostic prediction.

As far as we know, this study is the first to construct a pathology *T*-score prediction model in IgA nephropathy. At the same time, it is also the first study to use a machine learning algorithm to identify clinical variables that may influence the development of tubular atrophy/interstitial fibrosis, which may be useful for assessing the prognosis and targeted medication guidance. However, there is a limitation in our study. The model has been developed and tested in a single-center cohort of patients with IgAN; therefore, multicenter prospective cohort and ethnic-based cohort studies are necessary, which will further confirm the reliability of the pathology *T*-score prediction model, expand the scope of application of the model, and provide possibilities for clinical application.

In conclusion, our pathology *T*-score prediction (*T*_pre_) model is a reliable tool for predicting the presence or absence of pathological T lesions. At the same time, it can also be used to assist clinicians in predicting the prognosis of patients with IgAN. A prospective multicenter cohort study is necessary to explore the potential value and robustness of this T prediction tool in the management of IgA nephropathy.

## Data availability statement

The data presented in the study are deposited in the GitHub repository (https://github.com/zhangd17-web/IGAN_MI).

## Author contributions

Research idea and study design: HZ, X-JZ, LW, DZ, and LX. Data acquisition: LX, SS, X-JZ, and HZ. Data analysis/interpretation: LX, DZ, LW, HW, and X-JZ. Statistical analysis: LX and DZ. Supervision or mentorship: X-JZ, HZ, HW, LW, RC, GC, LL, SS, XZ, SH, LD, and JL. Each author contributed important intellectual content during manuscript drafting or revision and agrees to be personally accountable for the individual’s own contributions and to ensure that questions pertaining to the accuracy or integrity of any portion of the work, even one in which the author was not directly involved, are appropriately investigated and resolved, with documentation in the literature if appropriate.
